# Arrhythmogenic mechanisms in ryanodine receptor channelopathies

**DOI:** 10.1007/s11427-014-4778-z

**Published:** 2014-12-05

**Authors:** Yan-Ting ZHAO, Carmen R. VALDIVIA, Georgina B. GURROLA, Jonathan J. HERNÁNDEZ, Héctor H. VALDIVIA

**Affiliations:** Center for Arrhythmia Research, Department of Internal Medicine, Cardiovascular Division, University of Michigan, Ann Arbor, MI 48109, USA

**Keywords:** ryanodine receptors, CPVT, sarcoplasmic reticulum, cardiac arrhythmias

## Abstract

Ryanodine receptors (RyRs) are the calcium release channels of sarcoplasmic reticulum (SR) that provide the majority of cal-cium ions (Ca^2+^) necessary to induce contraction of cardiac and skeletal muscle cells. In their intracellular environment, RyR channels are regulated by a variety of cytosolic and luminal factors so that their output signal (Ca^2+^) induces finely-graded cell contraction without igniting cellular processes that may lead to aberrant electrical activity (ventricular arrhythmias) or cellular remodeling. The importance of RyR dysfunction has been recently highlighted with the demonstration that point mutations in *RYR2*, the gene encoding for the cardiac isoform of the RyR (RyR2), are associated with catecholaminergic polymorphic ventricular tachycardia (CPVT), an arrhythmogenic syndrome characterized by the development of adrenergically-mediated ventricular tachycardia in individuals with an apparently normal heart. Here we summarize the state of the field in regards to the main arrhythmogenic mechanisms triggered by RyR2 channels harboring mutations linked to CPVT. Most CPVT mutations characterized to date endow RyR2 channels with a gain of function, resulting in hyperactive channels that release Ca^2+^ spontaneously, especially during diastole. The spontaneous Ca^2+^ release is extruded by the electrogenic Na^+^/Ca^2+^ exchanger, which depolarizes the external membrane (delayed afterdepolarization or DAD) and may trigger untimely action potentials. However, a rare set of CPVT mutations yield RyR2 channels that are intrinsically hypo-active and hypo-responsive to stimuli, and it is unclear whether these channels release Ca^2+^ spontaneously during diastole. We discuss novel cellular mechanisms that appear more suitable to explain ventricular arrhythmias due to RyR2 loss-of-function mutations.

Ca^2+^ is an indispensable factor for the generation of the contractile force that gives rise to the heartbeat. In ventricular cardiomyocytes, depolarization of the sarcolemma and its invaginations, the T-tubules, induces the opening of voltage-dependent L-type Ca^2+^ channels (also known as dihydropyridine receptors, DHPRs), which permit the entry of a small amount of Ca^2+^ (*I*_Ca_) into the cell. In adult cardiomyocytes, *I*_Ca_ is insufficient to elicit full contractions, but is capable of triggering massive release of Ca^2+^ from the sarcoplasmic reticulum (SR), which then bathes the contractile filaments, binds to Ca^2+^-binding proteins, and finally induces contraction [1]. This amplification process, known as Ca^2+^-induced Ca^2+^ release (CICR) [2], is mediated by Ca^2+^ activation of Ca^2+^ release channels (also known as ryanodine receptors, RyRs), which represent the major, if not the only, pathway for Ca^2+^ release from the SR [3]. CICR amplifies the incoming Ca^2+^ signal ~4 20 fold (depending on the animal species) and is therefore the major component of the intracellular Ca^2+^ transient that induces contraction. The efficiency of communication between T-tubules and the SR, or specifically, the “fidelity” of DHPR→RyR signaling, determines to a great extent the magnitude of Ca^2+^ release from the SR, and thus the force of contraction [4].

*I*_Ca_ is a critical component of the plateau phase of the action potential (AP) and it has been experimentally established that maneuvers that alter the AP waveform may directly or indirectly affect SR Ca release [5]. Thus, APs control the magnitude and time course of Ca^2+^ release, and the flow of information AP→Ca^2+^ release is unquestionable. However, there is also an emergent role for the retroactive feedback that SR Ca^2+^ release exerts on *I*_Ca_ (Ca^2+^ release→ AP). L-type Ca^2+^ channels open in response to depolarization and then enter into an absorbing state of inactivation, which comprises a fast, Ca^2+^-dependent component, and a slow, voltage-dependent component [6]. Hence, in the absence of Ca^2+^ release, Ca^2+^-dependent inactivation of *I*_Ca_ is slowed, which prolongs the duration of the AP and favors the generation of arrhythmic substrates [7]. Also, Ca^2+^ released from the SR is normally recaptured by the SR Ca^2+^ ATPase (SERCA2a), but it may also be rapidly extruded by the electrogenic Na^+^/Ca^2+^ exchanger of the sarcolemma, generating in turn an inward current that may bring membrane potential to threshold and generate an untimely AP. Therefore, there is mutual entrainment of APs and Ca^2+^ release, and it thus follows that RyR dysfunction, by altering Ca^2+^ release, prompts cardiomyocytes to generate arrhythmic behavior. In this review we discuss conventional and novel pathogenic mechanisms by which RyR dysfunction may cause cardiac arrhythmias.

## 1 Catecholaminergic polymorphic ventricular tachycardia

### 1.1 Brief clinical description

Catecholaminergic polymorphic ventricular tachycardia (CPVT) is a genetic and highly malignant arrhythmogenic disease that may lead to sudden death in children and young adults [8]. To date, more than ~160 mutations in *RYR2*, the gene encoding for the cardiac isoform of the RyR (RyR2), are linked to CPVT1, the dominant form of the syndrome, but it is clear that CPVT is an arrhythmogenic syndrome caused by intracellular Ca^2+^ mishandling, as mutations in cardiac isoform of the calsequestrin (CSQ2) [9] and triadin [10], two SR luminal proteins that regulate Ca^2+^ release through RyR2 channels, cause a recessive form of the disease (CPVT2 and CPVT3, respectively). Very recently, mutations in calmodulin, which directly binds to the cytosolic portion of RyR2, were also linked to this syndrome [11]. When CPVT was first described by Leenhardt et al. [12] in 1995, it was considered a rare syndrome, but molecular screenings for *RYR2* and *CSQ2* mutations in children with positive exercise stress testing and absence of cardiac structural defects have improved diagnosis and prognosis of the disease. As the name implies, CPVT is triggered by the surge of catecholamines that usually occurs during physical exercise or emotional stress in otherwise electrophysiologically normal individuals. Although electrocardiography (ECG) abnormalities are diverse (hence the term “polymorphic”), many patients present bidirectional ventricular tachycardia (BiVT), a 180° beat-to-beat rotation of the axis of the QRS complexes that is considered a cardinal sign of the disease [13]. Remarkably, BiVT is also the hallmark of ventricular tachycardias in patients with digitalis intoxication [14], which arise from Na^+^-induced Ca^2+^ overload, clearly linking both syndromes to common intracellular Ca^2+^ mishandling mechanisms.

### 1.2 Conventional arrhythmogenic mechanism for CPVT

**Figure 1:**
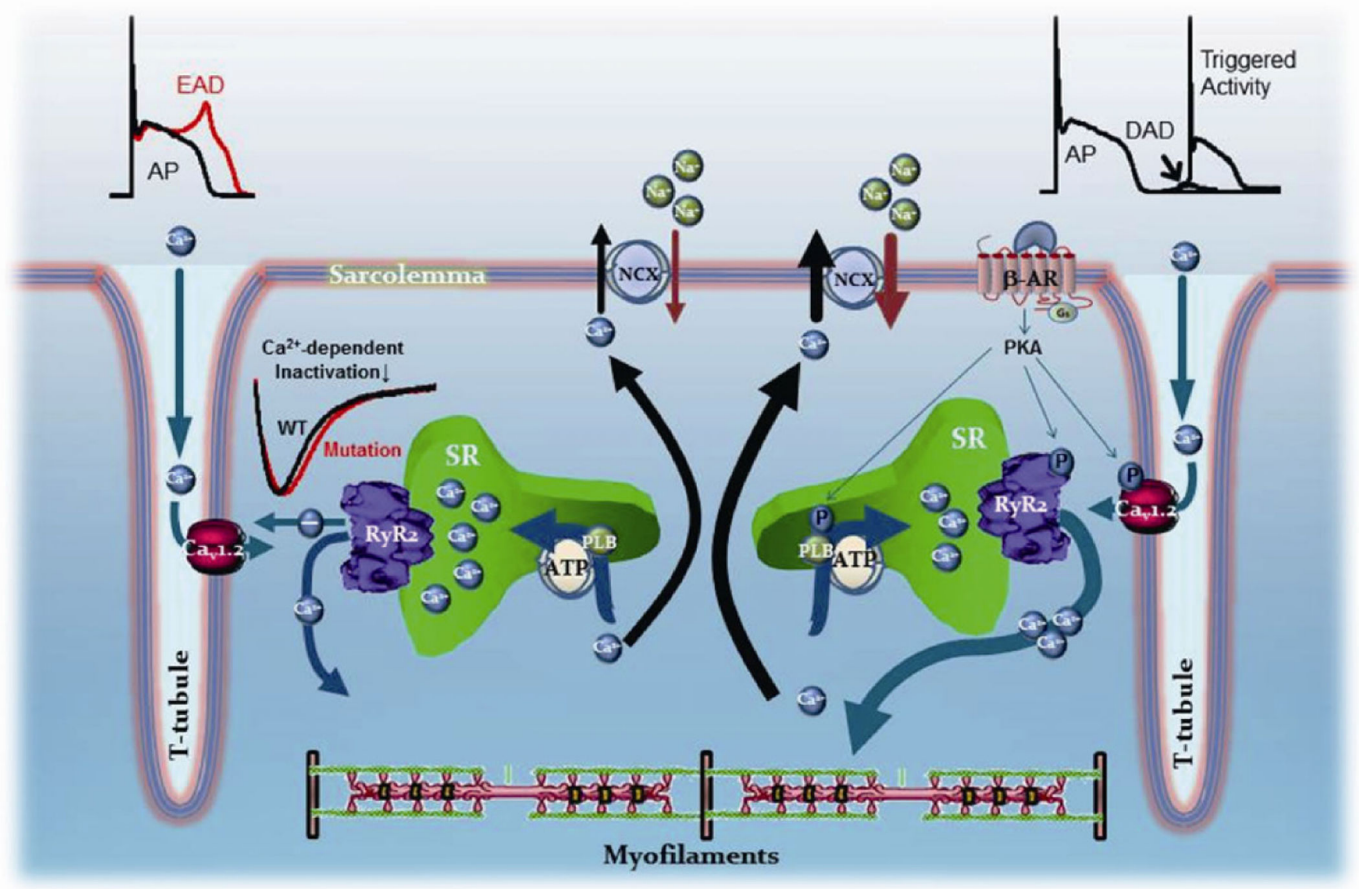
Arrhyhmogenic mechanisms in ventricular myocytes harboring RyR2 mutations linked to CPVT. Left side, CPVT mutations that render RyR2 channels hypo-active or hypo-responsive to stimuli. Depolarization of T-tubules open L-type Ca^2+^ channels (DHPRs, or Ca_v_1.2), which induce the opening of RyR2 channels in the SR. Ca^2+^ release from the SR then contributes to inactivate the L-type Ca^2+^ channel current (*I*_Ca_). If Ca^2+^ release is reduced, then there is altered inactivation of *I*_Ca_, prolongation of the action potential, and generation of early afterdepolarizations (EADs). Right side, The vast majority of CPVT mutations endow RyR2 channels with a gain-of-function. Stimulation of β_1_-adrenergic receptors by epinephrine activates PKA, which enhances Ca^2+^ entry and accelerates SR Ca^2+^ reuptake by phosphorylating L-type Ca^2+^ channels and phospholamban, respectively. The overall effect of sympathetic stimulation is an intracellular Ca^2+^ overload that increases the strength of contractions. In the process, when SR Ca^2+^ content is elevated, RyR2 channels hyper-sensitized by the CPVT mutations release Ca^2+^ spontaneously, usually in diastole, generating a depolarizing current (delayed afterdepolarization or DAD) as the released Ca^2+^ is extruded by the electrogenic Na^+^/Ca^2+^ exchanger. If Ca^2+^ released is of sufficient mass, it may generate a full action potential, which is the basis for triggered activity. See text for more details.

The vast majority of the RyR2-linked CPVT mutations studied to date have been found to endow RyR2 channels with a gain of function, that is, the mutations generate hyper-active or hyper-reactive RyR2 channels, and this finding restricts the cellular basis of the cardiac arrhythmias to a well-defined cascade of events, succinctly described as follows: during sympathetic stimulation, enhanced *I*_Ca_ plus enhanced SR Ca^2+^ uptake promoted by activation of PKA and likely CaMKII, lead to intracellular Ca^2+^ overload. In normal ventricular myocytes, this excess of intracellular [Ca^2+^] promotes stronger contractions (increased inotropism), a characteristic benefit of the fight-or-flight response. But in the setting of CPVT, this Ca^2+^ overload constitutes a powerful incentive for a group of mutation-deranged hyperactive RyR2 channels to release Ca^2+^ on their own, that is, to spew Ca^2+^ in the absence of *I*_Ca_ stimulation. When spontaneous Ca^2+^ release occurs during diastole, Ca^2+^ is rapidly extruded by the Na^+^/Ca^2+^ exchanger, as mentioned above, generating an inward current and gradually depolarizing the membrane potential, a phenomenon termed “delayed afterdepolarization”, or DAD (Figure 1, right side). If the amount of Ca^2+^ release is of sufficient mass to bring a DAD to threshold, it generates a full AP, which is the cellular basis for the triggered activity that disseminates to contiguous cells, forming discrete ectopic foci. In this scheme, therefore, spontaneous Ca^2+^ release via hyperactive RyR2 channels entrains sarcolemmal currents by means of the Na^+^/Ca^2+^ exchanger at a well-defined period of the AP (phase 4 or diastole). Note also that, although hyperactive, mutant RyR2 channels are not portrayed malfunctioning during the systolic phase of Ca^2+^ release. Most studies that used mice as models of CPVT remark the excessive diastolic Ca^2+^ leak [15 17], but do not observe significant alterations in their systolic [Ca^2+^]_i_ transient.

### 1.3 Molecular mechanisms that may underlie excessive Ca^2+^ release

Several molecular mechanisms have been advanced to explain why RyR2 channels bearing CPVT mutations are prone to discharge excessive amounts of Ca^2+^ during diastole and cause DADs. A reduction in the threshold for activation of mutant RyR2 channels by luminal Ca^2+^ is one of the most prominent mechanisms [18,19]. In this scheme, normal RyR2 channels do not release Ca^2+^ on their own because the luminal [Ca^2+^] that is reached during basal conditions and even during sympathetic stimulation is below their threshold for activation. In contrast, a CPVT mutation presumably lowers the RyR2 channel’s threshold for luminal Ca^2+^ activation, and although mutant channels remain “silent” under basal conditions, they “ignite” during the normal sympathetic Ca^2+^ overload. This scheme has been demonstrated in a great number of, but not all, RyR2 mutations linked to CPVT [15,16], and although intelligible at its core, is likely accompanied by other mechanisms. Defective RyR2 inter-domain interactions was originally proposed by Ikemoto’s group to explain the effect of synthetic peptides with amino acid sequence identical to specific segments of the RyR to increase the activity of the same channels [20]. In this scheme, normal RyR2 channels are believed to be leak-resistant by interdomain interactions that “zip” various segments of the RyR2 protein and stabilize the channel in a closed state. Consequently, mutations occurring within the interactive domains may trigger CPVT episodes because they tend to ‘‘unzip’’ those interactions and thus destabilize the channel, favoring Ca^2+^ leak [21]. This hypothesis is supported by the fact that most CPVT mutations fall within three well defined segments (or “hot spots”) of the RyR2 linear sequence, namely, residues 77 466 (N-terminal segment or domain I), residues 2246 2534 (domain II), and residues 3778 4959 (C-terminal segment or domain III). It is therefore conceivable that these segments interact with each other to stabilize the channel in the closed state and, in fact, there is already some structural evidence that domains I and II do so [22]. Mutations in these regions would thus destabilize the close state of the channel and allow excessive Ca^2+^ leak. The hierarchy of these “hot spots” in the control of the channel’s stability is also demonstrated by the fact that equivalent mutations in *RYR1*, the gene encoding for the skeletal isoform of the RyR (RyR1), give rise to Malignant Hyperthermia and Central Core Disease, two devastating diseases of skeletal muscle whose primary pathogenic mechanism is also excessive Ca^2+^ leak from the SR [23]. Dissociation of FKBP12.6 from RyR2 has also been postulated as the central mechanism that triggers CPVT [24–26]. Marks and colleagues have found that RyR2 channels harboring mutations that give rise to CPVT tend to dissociate FKBP12.6 more readily than wild-type channels. Since FKBP12.6 is presumed to stabilize RyR2 channels in their closed state (a contentious issue by itself [27,28]), a FKBP12.6-free RyR2 channel would dwell in the open state for longer times, generating the excessive Ca^2+^ leak which is at the center of the pathogenic mechanisms of CPVT mentioned above. This hypothesis is attractive because CPVT episodes occur by definition during adrenergic stimulation, and normal phosphorylation of RyR2 channels by PKA (the main kinase linking β-adrenergic stimulation to cellular effects) is presumed to dissociate FKBP12.6 from RyR2 [29]. Thus, it would only be required that this reaction was more pronounced in the mutant RyR2 channels to generate excessive Ca^2+^ leak. However, several key aspects of this hypothesis have not been confirmed by others, since others have found no evidence of FKBP12.6 dissociation in CPVT mutation-harboring RyR2 [30]. Finally, it is worth considering that there is no a priori reason to rule out multiple mechanisms affected by a single mutation and working in unison to destabilize the channel and generate spontaneous Ca^2+^ release. This heterogeneity of RyR2 dysfunction caused by CPVT mutations is actually expected if we consider that most CPVT mutations fall in domains of the RyR2 sequence that control several aspects of channel function, including regions involved in Ca^2+^ sensing, excitation-contraction coupling, phosphorylation, redox sensing, and others (reviewed in [30]). Also, some amino acid residues appear central to maintain the global integrity of the channel, and mutations in these critical residues alter multiple aspects of channel function. Loaiza et al. [31] showed at least three different mechanisms of channel dysfunction in RyR2-V2475F channels.

### 1.4 Different arrhythmogenic mechanisms for RyR2 loss-of-function mutations linked to CPVT?

Although spontaneous Ca^2+^ release, especially during diastole, is the most accepted cellular mechanism underlying ventricular tachyarrhythmias in CPVT, it appears insufficient to explain the same tachyarrhythmias in patients suffering RyR2 loss-of-function mutations. If a mutation renders a RyR2 channel hypo-active or hypo-responsive, is it still possible that this channel “ignites” eagerly during a normal, sympathetically-induced Ca^2+^ overload? If the hallmark of a hyperactive RyR2 channel (gain-of-function mutation) is its trigger-readiness and promptitude to release Ca^2+^ on its own, would a hypo-active channel show exact opposite attributes, that is, resilience to release Ca^2+^ spontaneously, if at all? The question is relevant because, scanning the phenotype of several RyR2 channels harboring CPVT mutations, Chen and co-workers [32] found that at least one of them, RyR2-A4860G, exhibited a pronounced loss-of-function characterized by a markedly depressed response to luminal Ca^2+^ and extremely low open probability in the presence of full agonists such as cytosolic Ca^2+^ and caffeine. In a cohort of CPVT patients, the RyR2-A4860G mutation had been detected in a 6-year-old girl who presented idiopathic ventricular fibrillation, was treated with β-blockers, and had an implantable cardioverter [33].

The experiments of Chen and colleagues were crucial to expose the novel loss of function attribute of RyR2-A4860G but could not reveal the mechanism by which the mutation causes arrhythmias because they were conducted using homogeneous recombinant mutant protein (equivalent to a homozygous mutation, whereas the patient was heterozygous for the mutation) and in heterologous expression systems (HEK293 cells), which are poor substitutes for cardiac myocytes [32]. We generated a mouse line harboring the RyR2-A4860G mutation to investigate whether this animal recapitulated the cardinal signs of the disease and could yield information on its arrhythmogenic mechanisms [34]. Under basal conditions, mice heterozygous for the RyR2-A4860G mutation (RyR2-A4860G^+/−^ ) exhibited bradycardia but showed no major cardiac structural abnormalities. No homozygotes were detected at birth, indicating that the RyR2-A4860G mutation is phenotypically too strong to be harbored in the two alleles. Anesthetized RyR2-A4860G^+/−^ mice injected with an arrhythmogenic cocktail (120 mg kg^−1^ caffeine and 2 mg kg^−1^ epinephrine) displayed ECG alterations consisting with QRS alternans, premature ventricular complexes and BiVTs, whereas ECGs of WT mice remained normal under the same conditions. Simultaneous recording of action potentials and intracellular calcium transients ([Ca^2+^]_i_) in isoproterenol (300 nmol L^−1^)-stimulated RyR2-A4860G^+/−^ ventricular myocytes showed prolonged action potentials and interspaced bursts of altered Ca^2+^ release consisting of a normal peak followed by a second, prolonged phase of release during the systolic phase. Remarkably, early afterdepolarizations (EADs) were observed only during the prolonged phase of release, and were abolished by either the NCX inhibitor CB-DMB extracellularly applied or the Ca^2+^ chelator EGTA (10 mmol L^−1^, intracellular). The RyR2-A4860G loss-of-function mutation thus reveals novel mechanisms of arrhythmogenesis in CPVT: first, mutant RyR2 channels decrease the peak of Ca^2+^ release during systole and thus impair L-type Ca^2+^ channel inactivation (faulty retroactive Ca^2+^ release→AP feedback), both of which gradually overload the SR. The resultant SR overload then causes bursts of prolonged Ca^2+^ release, which activate electrogenic Na^+^/Ca^2+^ exchange activity during the plateau and descending phases of the AP, triggering EADs (Figure 1, left side). These novel pathways by which RyR2 channels engage membrane currents may also produce life-threatening arrhythmias.
